# Supplementary material for: Dynamics of Ex Vivo Mesenchymal Stromal Cell Potency under Continuous Perfusion
Source: Int J Mol Sci. 2023 May 31;24(11):9602. doi: 10.3390/ijms24119602 (PMC10253999; doi:10.3390/ijms24119602)
Supplement: Supplementary file 1 [file ijms-24-09602-s001.zip › ijms-2400605-supplementary.pdf]

	MSC Viability	
	1M	3M
Serum media	72.7%	65.9%
Exosome-depleted serum media	70.8%	66.5%
Serum-free media	43.2%	39.7%

Table S1: Comparing MSC viability after perfusion with serum media, exosome-depleted serum media, and serum-free media

	Baseline Perfusion		Activated Perfusion		Prelicensed Perfusion	
	Yield	Viability	Yield	Viability	Yield	Viability
3M	5.04e5	68.3%	1.56e6	62.5%	1.02e6	71.4%
3M	4.89e5	65.9%	1.94e6	68.0%	1.37e6	76.9%
1M	4.79e5	72.7%				
1M	5.29e5	70.8%				

Table S2: Yield and viability of MSCs harvested from n=2 bioreactors after 72h perfusion via Accutase (Sigma Aldrich, St. Louis, MO) after 72-hour perfusion

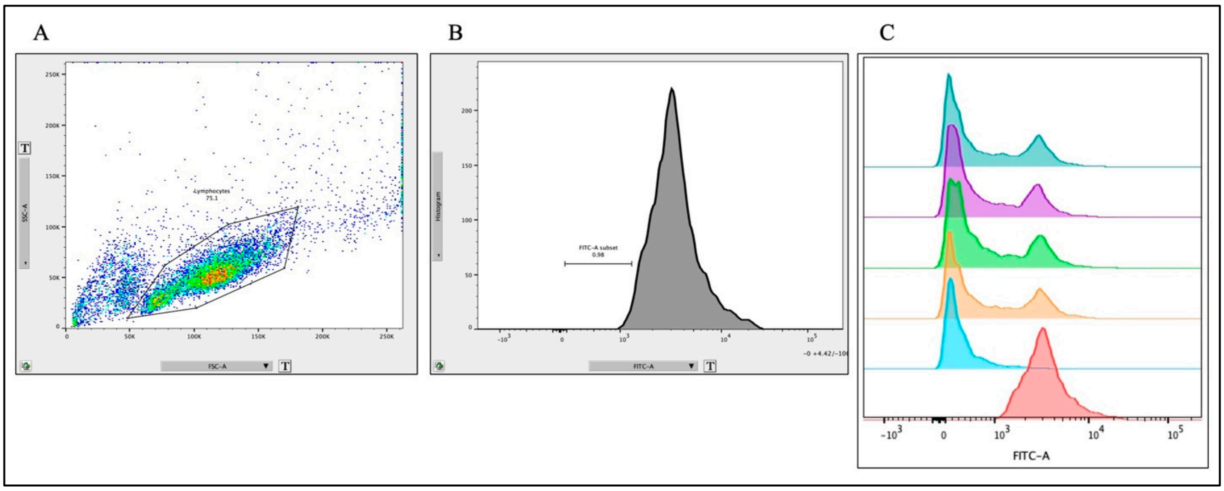

Figure S1: FlowJo Representative Figures and Histograms. **(A)** Gating strategy for lymphocyte selection. **(B)** Proliferative gate set with from unstimulated FITC-stained lymphocytes. **(C)** Histograms showing differences in percent proliferation between controls and fractions (bottom to top: unstimulated control, stimulated control, 4 representative fractions).

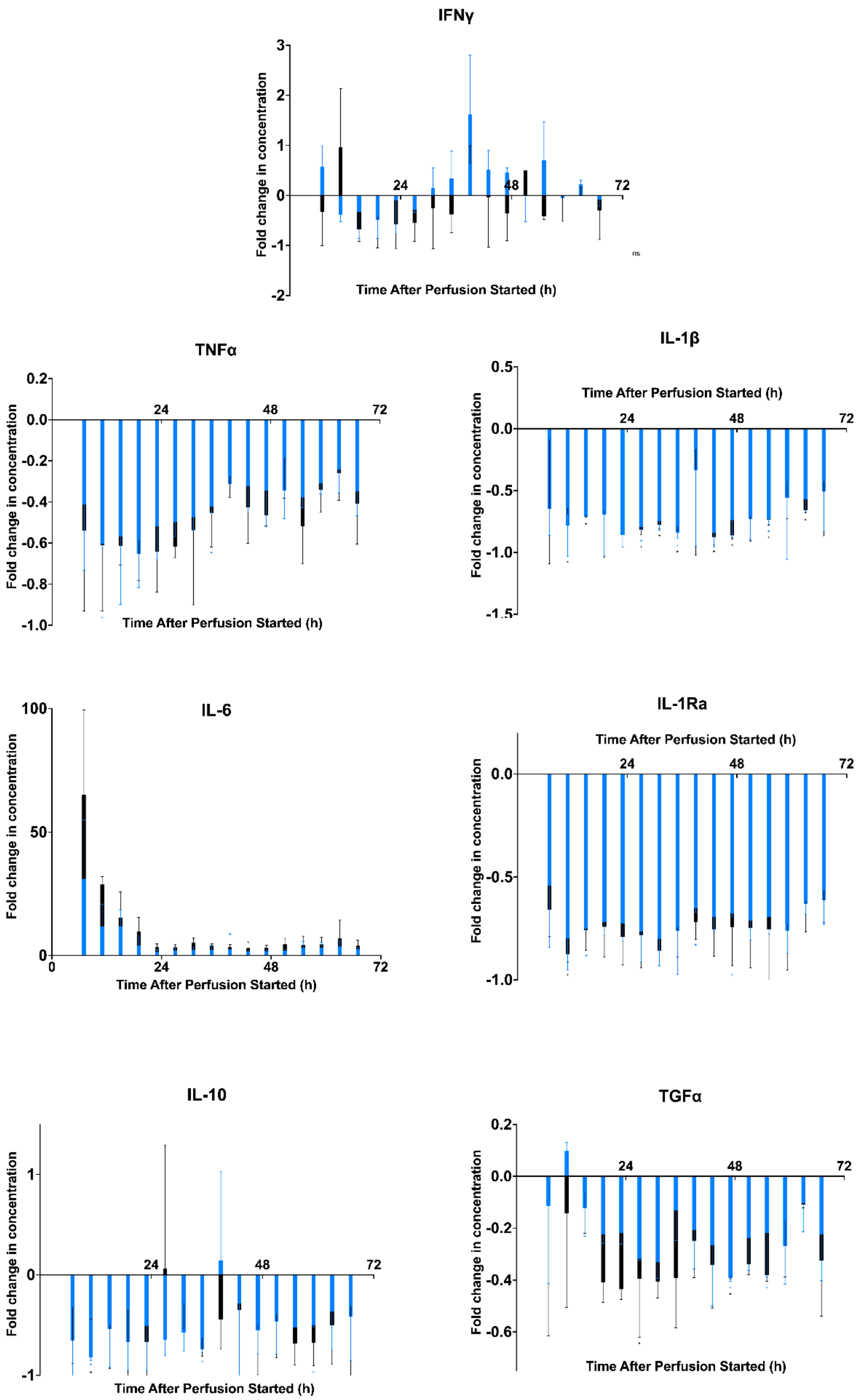

Figure S2: Cytokines analyzed from baseline perfusion potency assay

Time After Perfusion (h)		TGFα	IFNγ	IL-10	Il-1Ra	IL-1ß	IL-6	TNF-α
	1M vs. 3M	>0.9999	0.4323	0.7863	0.8824	0.7734	0.5866	0.9329
7	1M vs. 0M	0.9522	0.4364	0.3465	0.3539	0.2145	0.4575	0.4669
	3M vs. 0M	0.8726	0.809	0.7612	0.1349	0.9915	0.3343	0.4398
11	1M vs. 3M	0.7147	0.5071	0.0453	0.8107	0.7643	0.3093	0.9998
	1M vs. 0M	0.8639	0.2478	0.022	0.1276	0.2103	0.4528	0.0214
	3M vs. 0M	0.2191	0.6371	0.0211	0.0251	0.0469	0.0738	0.3345
15	1M vs. 3M	0.9065	0.7441	0.994	0.9953		0.9175	0.9789
	1M vs. 0M	0.8567	0.7321	0.4287	0.0211		0.3506	0.3676
	3M vs. 0M	0.5161	0.2426	0.5102	0.0895	0.0211	0.4123	0.1009
19	1M vs. 3M	0.2199	0.9933	0.8397	0.9773	0.9962	0.5612	0.9329
	1M vs. 0M	0.1249	0.458	0.2799	0.0606	0.308	0.5616	0.1676
	3M vs. 0M	0.1061	0.6937	0.8005	0.1338	0.3556	0.3628	0.2196
23	1M vs. 3M	0.0653	0.7255	0.9169	0.8507	>0.9999	0.3418	0.7927
	1M vs. 0M	0.0609	0.9785	0.5156	0.08	0.0786	0.373	0.2071
	3M vs. 0M	0.1359	0.4831	0.331	0.1156	0.0786	0.2237	0.2008
27	1M vs. 3M	0.8904	0.692	0.7583	0.993	0.8596	0.5958	0.3422
	1M vs. 0M	0.3576	0.1078	0.1591	0.1309	0.0211	0.4866	0.0932
	3M vs. 0M	0.0211	0.4153	0.9969	0.139	0.0307	0.2012	0.0566
31	1M vs. 3M	0.5652	0.8245	0.508	0.8636	0.848	0.3794	0.9715
	1M vs. 0M	0.1041	0.8672	0.2071	0.1085	0.0664	0.3426	0.0909
	3M vs. 0M	0.1266	0.909	0.5113	0.0568	0.022	0.2456	0.4189
35	1M vs. 3M	0.4342	0.4451	0.7258	0.9953	0.491	0.5408	0.9579
	1M vs. 0M	0.3169	0.7294	0.0236	0.1859	0.027	0.4737	0.0464
	3M vs. 0M	0.5113	0.5486	0.188	0.1301	0.0223	0.0975	0.2351
39	1M vs. 3M	0.9569	0.6323	0.718	0.6703	0.9723	0.5023	
	1M vs. 0M	0.3615	0.4415	0.9712	0.0211	0.7774	0.0211	
	3M vs. 0M	0.4442	0.3358	0.4137	0.0807	0.9608	0.1635	0.244
43	1M vs. 3M	0.9305	0.7805	0.9948	0.86	0.9175	0.321	0.7949
	1M vs. 0M	0.3136	0.4516	0.8678	0.0616	0.0917	0.2493	0.2608
	3M vs. 0M	0.5131	0.9982	0.7848	0.1194	0.0444	0.0399	0.2649
47	1M vs. 3M	0.7184	0.4102	0.988	0.8887	0.6151	0.4767	0.7131
	1M vs. 0M	0.0213	0.1435	0.2823	0.0212	0.129	0.1627	0.3268
	3M vs. 0M	0.0211	0.7195	0.5687	0.1702	0.0221	0.2309	0.0752
51	1M vs. 3M	0.6397		0.9831	0.9703	0.9926	0.522	0.6842
	1M vs. 0M	0.0787	>0.9999	0.4243	0.0926	0.1518	0.1971	0.2634
	3M vs. 0M	0.3355		0.5895	0.1741	0.1834	0.336	0.5727
55	1M vs. 3M	0.5765	0.4176	0.6242	0.9536	0.9963	0.9043	0.6699
	1M vs. 0M	0.0414	0.5926	0.926	0.0804	0.0342	0.4898	0.0876
	3M vs. 0M	0.4624	0.1024	0.2044	0.235	0.0397	0.4685	0.2345
59	1M vs. 3M	0.8622		0.6538	0.9853	0.9437	0.8826	0.9259
	1M vs. 0M	0.6652		0.0253	0.0986	0.5148	0.4385	0.1136

	3M vs. 0M	0.3454	0.9997	0.2206	0.2023	0.4375	0.4221	0.2118
63	1M vs. 3M	0.998	0.4143	0.9337	0.9939	0.6191	0.842	0.9882
	1M vs. 0M	0.0891	0.2344	0.5804	0.0539	0.1193	0.4236	0.3031
	3M vs. 0M	0.5867	0.9999	0.4635	0.1603	0.0214	0.5932	0.3268
67	1M vs. 3M	0.8762	0.8808	0.9841	0.9365	0.9752	0.6895	0.9271
	1M vs. 0M	0.4082	0.7829	0.5809	0.1289	0.3927	0.2393	0.2281
	3M vs. 0M	0.4705	0.8	0.832	0.183	0.5706	0.3358	0.3081

Table S3: p-values from multiple unpaired student's t-test comparing cytokine secretions in cocultures with fractions from 3 M, 1 M, and 0 M acellular control in baseline perfusion.

Time After Perfusion (h)	p-values	
	Inflamed Perfusion	Prelicensed Perfusion
1	0.000813	0.003915
3	0.004642	0.003864
5	0.295765	0.000467
7	0.000384	0.005096
9	0.00313	0.000596
11	0.013266	0.003204
13	0.00875	0.008016
15	0.298488	0.006995
17	0.005008	0.001701
19	0.078466	0.000294
21	0.034827	0.00311
23	0.547231	0.000632
25	0.015024	0.037852
27	0.015058	0.000221
29	0.040048	0.00641
31	0.000939	<0.000001
33	0.016319	0.001946
35	0.003877	0.007466
37	0.001703	0.004262
39	0.000135	0.01306
41	0.007751	0.007984
43	0.064586	0.002277
45	0.107832	0.00187
47	0.07936	0.001989
49	0.146812	0.002079
51	0.024674	0.003636
53	0.337186	0.001121
55	0.023983	0.007027
57	0.029411	0.001492
59	0.006614	0.005578
61	0.040758	0.001378
63	0.002682	0.004872
65	0.258344	0.000368
67	0.003859	0.010858

Table S4: p-values from multiple unpaired student's t-test comparing potency of fractions 3M to 0M acellular control in both inflamed and prelicensed perfusions

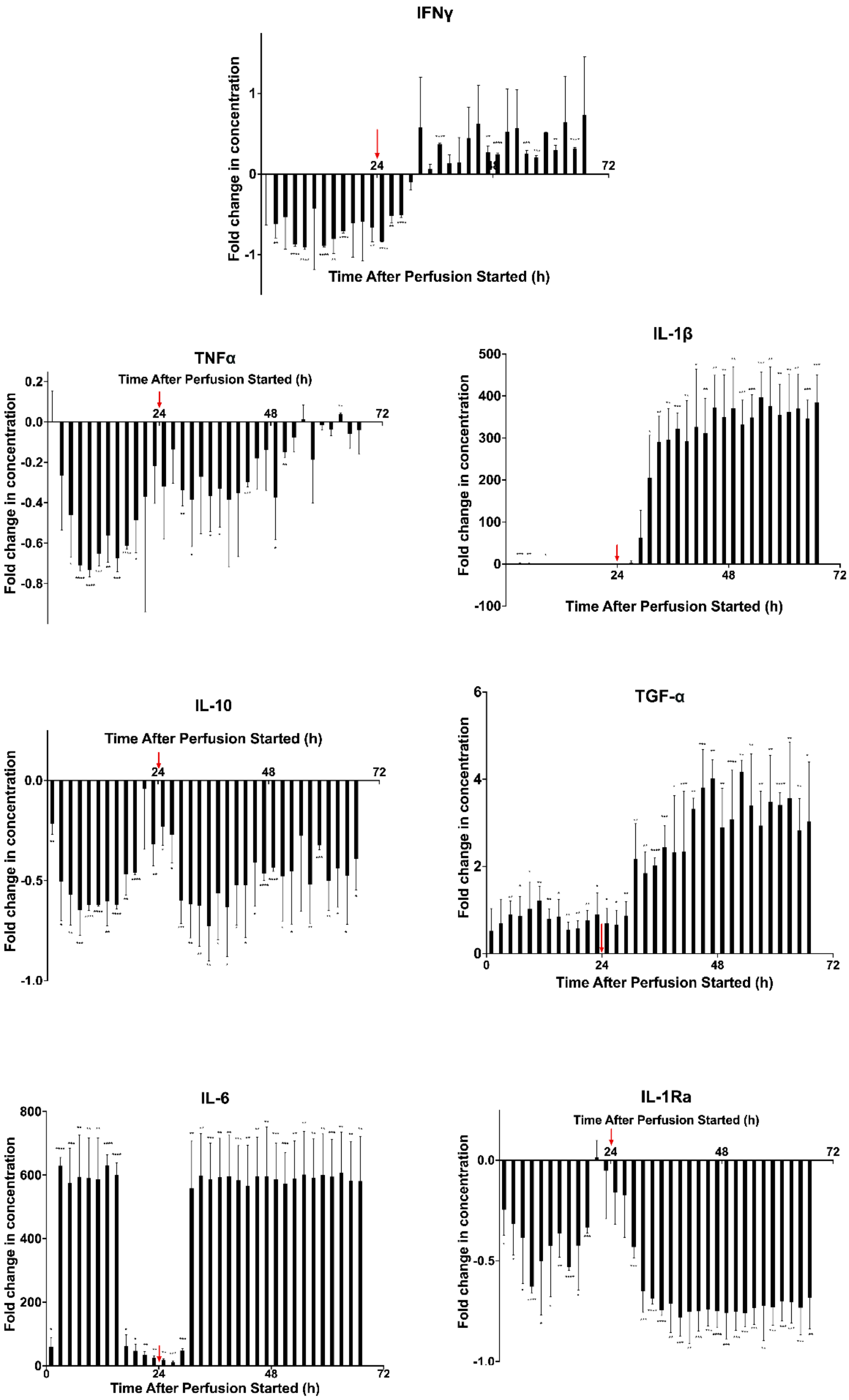

Figure S3: Cytokines analyzed from inflamed perfusion potency assay using 3 M cell dosage

Time After Perfusion (h)	TGFα	IFNγ	IL-10	Il-1Ra	IL-1ß	IL-6	TNF-α
1	0.150661	0.998704	0.004767	0.0353	0.403698	0.02375	0.998355
3	0.086919	0.006111	0.01131	0.029881	0.000153	0.000024	0.167188
5	0.007421	0.081905	0.002825	0.042609	0.001973	0.000797	0.01786
7	0.027517	0.000006	0.000893	0.00004	0.115151	0.001465	0.000018
9	0.044085	0.000011	0.000003	0.031718	0.011455	0.001245	0.000028
11	0.003046	0.378456	<0.000001	0.043547	0.76663	0.001417	0.000279
13	0.004244	0.000002	0.001021	0.00606	0.77341	0.000046	0.001773
15	0.019838	0.003402	0.000014	0.000008	0.82107	0.000076	0.000292
17	0.005293	0.000012	0.001394	0.03602	0.336614	0.042111	0.000006
19	0.003835	0.073856	<0.000001	0.000171	0.857734	0.017933	0.010585
21	0.004605	0.105768	0.805953	0.780221	0.600995	0.003182	0.45229
23	0.036751	0.005939	0.007463	0.72014	0.146356	0.002749	0.110611
25	0.024767	<0.000001	0.019316	0.152138	0.532688	0.002873	0.099226
27	0.024247	0.001864	0.036066	0.22727	0.35072	0.000588	0.229258
29	0.009649	0.000038	0.000817	0.000164	0.178589	0.000208	0.001873
31	0.009992	0.147211	0.006005	0.00042	0.024038	0.002868	0.044564
33	0.00294	0.179789	0.006011	0.000002	0.001145	0.001466	0.172243
35	0.000048	0.095342	0.001905	<0.000001	0.00226	0.000883	0.022842
37	0.000941	0.000018	0.01388	0.001028	0.000123	0.001057	0.040508
39	0.035236	0.085173	0.010948	0.000128	0.006394	0.001286	0.113721
41	0.042398	0.424557	0.01023	0.001099	0.014506	0.000766	0.123903
43	0.000128	0.111568	0.025452	0.000193	0.002774	0.00152	0.000195
45	0.003716	0.085595	0.031288	0.000104	0.001115	0.00111	0.112358
47	0.000388	0.005568	0.000019	0.000079	0.003687	0.002738	0.178366
49	0.005103	0.000067	0.000026	0.000527	0.002909	0.000928	0.035402
51	0.009098	0.167303	0.021156	0.00014	0.000589	0.000502	0.001461
53	0.000082	0.104153	0.040333	0.000041	0.000355	0.001011	0.132714

**55**	0.007708	0.000951	0.273547	0.000099	0.000336	0.00152	0.408
**57**	0.003019	0.000434	0.009481	0.001941	0.002229	0.001082	0.195187
**59**	0.004731	<0.000001	0.000113	0.000154	0.001138	0.001311	0.234997
**61**	0.000033	0.001946	0.004207	0.000256	0.002078	0.000916	0.110996
**63**	0.008553	0.119506	0.016961	0.000274	0.001481	0.001173	0.001763
**65**	0.00268	0.000014	0.026926	0.000715	0.000159	0.00118	0.211141
**67**	0.017821	0.152097	0.01214	0.001517	0.000522	0.001955	0.56982

Table S5: p-values from multiple unpaired student's t-test comparing cytokine secretions in cocultures with fractions 3M to 0M acellular control in inflamed perfusion

Time After Perfusion (h)	TGFα	IFNγ	IL-10	Il-1Ra	IL-1ß	IL-6	TNF-α
1	0.005555	0.077269	0.001584	0.118733	0.139199	0.10173	
3	0.000177	0.00002	0.234367	0.000578	0.032428	0.094421	0.002068
5	<0.000001	0.89284	<0.000001	0.000757	0.009219	0.100994	0.000392
7	0.007237	0.039236	0.003569	0.00037	0.350967	0.090643	0.009828
9	0.000016	0.497654	0.043189	0.004179	0.14571	0.166714	0.000495
11	0.022832	0.016853	0.006754	0.000004	0.314744	0.173093	0.014431
13	0.0068	0.690115	0.000004	0.000135	0.065716	0.069829	0.003015
15	0.001822	0.731507	0.00455	<0.000001	0.000004	0.005085	0.000242
17	0.128641	0.585038	0.009934	0.00005	0.018892	0.003303	0.036625
19	0.033026	0.808613	0.206608	0.000177	0.011629	0.007078	0.000147
21	0.019088	0.600717	0.000113	0.000019	0.000989	0.007667	0.004179
23	0.037314	0.463628	0.020518	0.000006	<0.000001	0.01693	0.009888
25	0.017717	0.033391	<0.000001	0.000021	<0.000001	0.016881	0.02517
27	0.000009	0.0232	0.015882	0.000075	0.00001	0.009064	0.039643
29	0.015392	0.086301	0.320096	0.000026	0.00092	0.015329	0.007046
31	0.016635	0.547506	0.033311	0.000209	0.000136	0.026678	0.002125
33	0.009125	0.110787	0.522473	0.000013	0.000517	0.027571	0.030371
35	0.000512	0.399927	0.016338	0.000055	0.00211	0.025288	0.625119

**37**	0.028116	0.506768	0.000633	0.00005	0.000061	0.019816	0.001416
**39**	0.001678	0.328186	0.002963	0.0001	0.00283	0.023384	0.09206
**41**	0.015792	0.322262	0.02792	0.000378	0.013246	0.036994	0.006721
**43**	0.003291	0.339657	0.054292	0.000121	0.019076	0.035203	0.000106
**45**	0.069552	0.306435	0.00823	0.000029	0.001536	0.020992	0.007523
**47**	0.091779	0.116814	0.042252	0.000011	0.026763	0.016291	0.729702
**49**	0.001684	0.123099	0.009335	<0.000001	0.000014	0.016149	0.541071
**51**	0.03633	0.273346	0.014902	0.000049	0.024825	0.017799	0.047543
**53**	0.001673	0.010772	<0.000001	0.000037	0.000756	0.018451	0.163925
**55**	0.002496	0.387969	0.065969	0.000037	0.006877	0.029766	0.040184
**57**	0.000512	0.777867	0.004279	0.000286	0.005546	0.029169	0.819038
**59**	0.000066	0.257934	0.000007	0.000016	0.001607	0.014279	0.128894
**61**	0.000006	0.581033	0.000762	0.009294	0.000031	0.02835	0.000009
**63**	0.051955	0.920623	0.156122	0.000203	0.061286	0.019429	0.00055
**65**	0.007184	0.40146	0.125512	0.001804	0.001601	0.016117	0.089647
**67**	0.002569	0.191089	0.115879	0.000265	0.063946	0.021706	0.000034
**69**		0.531473	0.145982	0.000216	<0.000001	0.023704	0.002684
**71**							0.06593

Table S6: p-values from multiple unpaired student's t-test comparing cytokine secretions in cocultures with fractions 3M to 0M acellular control in prelicensed perfusion

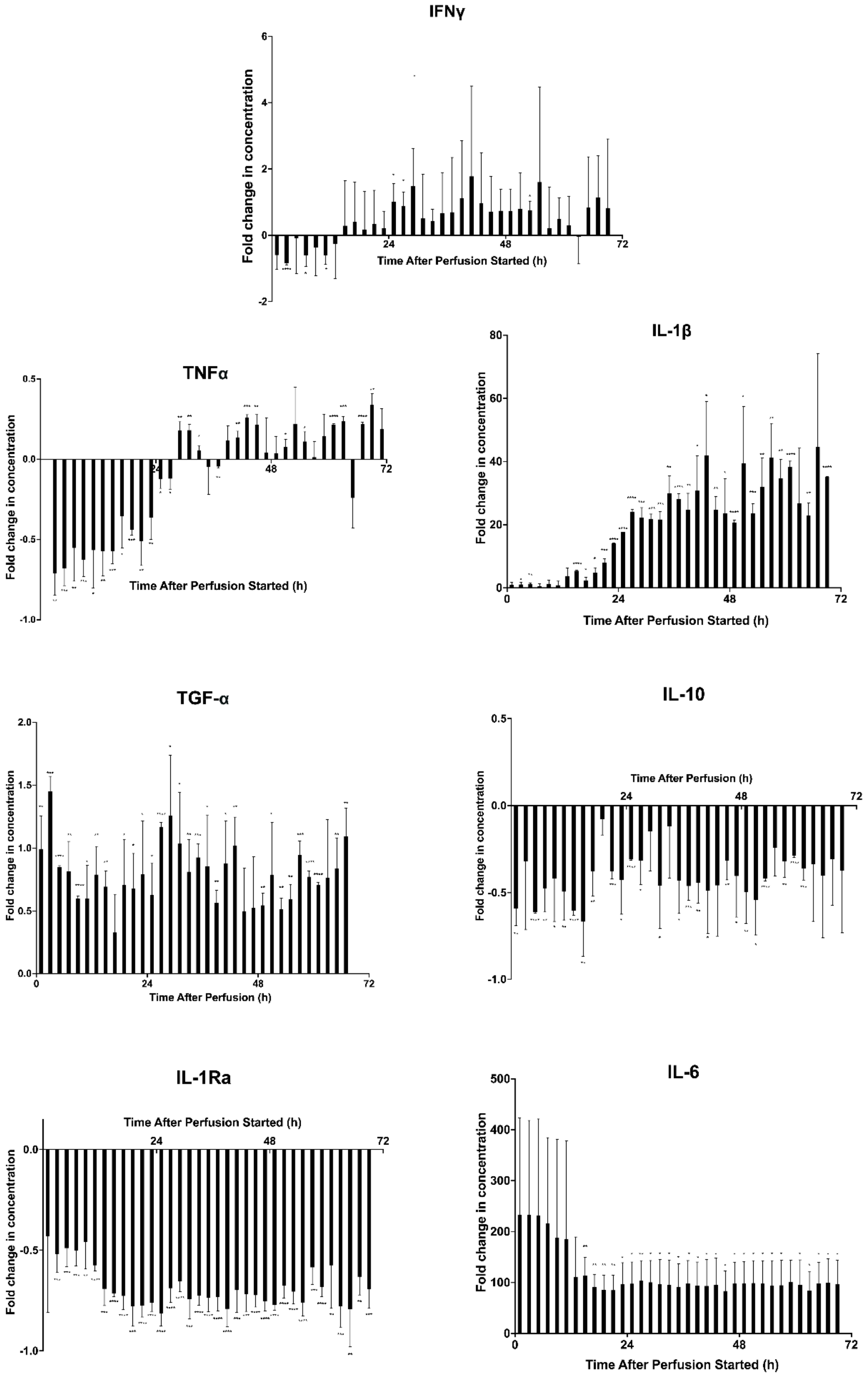
IFNγ
Fold change in concentration
Time After Perfusion Started (h)
TNFα
Fold change in concentration
Time After Perfusion Started (h)
IL-1β
Fold change in concentration
Time After Perfusion Started (h)
TGF-α
Fold change in concentration
Time After Perfusion (h)
IL-10
Fold change in concentration
Time After Perfusion (h)
IL-1Ra
Fold change in concentration
Time After Perfusion Started (h)
IL-6
Fold change in concentration
Time After Perfusion Started (h)

Figure S4: Cytokines analyzed from prelicensed perfusion potency assay using 3 M cell dosage
